# Milestones of Dentistry: Advent of Anesthetics in Oral Surgery

**DOI:** 10.3390/dj7040112

**Published:** 2019-12-10

**Authors:** Gabriele Cervino

**Affiliations:** Department of Biomedical and Dental Sciences, Morphological and Functional Images, University of Messina, Policlinico G. Martino, 98100 ME Via Consolare Valeria, Italy; gcervino@unime.it

**Keywords:** oral surgery, dentistry, pharmacology, history of medicine, anesthetics

## Abstract

The history of dentistry, of course, has followed a constant development since the dawn of society. The dental profession, reserved in ancient times to people with special skills and high rank, after the Middle Ages was diminished and practiced by barbers. The pharmacological evolution of oral surgery techniques has led this branch, today as never before, to obtain a level of specialization and preparation comparable to all other specialist medical branches. Some milestones in the history of dentistry will be considered so as to finally understand how the importance of anesthetic drugs was of primary importance, and which drugs are used today.

Since the beginning, within the various communities, men with special abilities, and knowledge about healing and aid played important roles in society. These people were the first to transfer and deposit medical knowledge [1].

The first known written evidence of a dentist is the tomb of Hesy-Re, an Egyptian who lived 4600 years ago and was described as “the best among those who treat teeth”. That Egypt was a land dedicaton to medicine and health; proof of dental care by a papyrus with the first known recipe of a toothpaste. In 1800 BC, the Code of Hammurabi refers to dental extractions imposed as a punishment. An Egyptian text, the Papyrus of Eber, speaks of dental diseases and various remedies for dental pain dating back to 1700 BC. Later, around 500–300 a.c [2,3], Hippocrates and Aristotle wrote about dentistry, including the eruption of teeth, the treatment of decayed teeth and gum disease, the extraction of teeth with forceps, and the use of wire to stabilize mobile teeth and fractures of the jaw. In 100 BC Celsus, a Roman medical writer, in his famous medical compendium “De Medicina” wrote about oral hygiene, the stabilization of mobile teeth, and the treatment of dental pain and jaw fractures [4]. The Etruscans, in the same period, practiced dental prosthesis using crowns and fixed bridges in gold [1].

Medical knowledge and practices, moreover, are reported during the Roman Empire history. The civilization and Middle Age advent led to a decrease in medical practices and to the medical knowledge loss. In the meantime, Chinese civilization saw a period of great development and prosperity In 700 AD, in Chinese civilization, treatments were so advanced that in a medical text the use of a metallic amalgam for the treatment of caries was described. During the Middle Ages, medicine was practiced by monks who had the best cultural education of that period. A medical text in China mentions the use of a silver compound, a prototype of the silver amalgam [1].

In Europe, after the year 1000, the demand for dentists was once again felt, so much so that in 1210 the first Barbers’ guild was created in France which had among its objectives the study and dissemination of methods of surgery. Barbers were divided into two groups: Surgeons who were educated and trained to perform complex surgical operations, and barber-surgeons who shaved and extract teeth [5,6].

In 1510, in Germany, the first dentistry book was printed by Artzney Buchlein, with practical advice for barber-surgeons on drilling, prostheses, and extractions. In 1563, Batolomeo Eustachi published the first accurate book on dental anatomy ‘Libellus de dentibus’. Just over 200 years later, in 1723, during the Enlightenment era, the French Pierre Fauchard guaranteed himself the title of “Father of modern dentistry” by writing the book “Dental surgery”, the first text to describe a complete system for dental practice, it was about surgery, pathology, anatomy [7,8,9]. In 1801, Richard C. Skinner wrote “Treatise on the Human Teeth”, the first dental book to be published in America. Robert Arthur originated the cohesive gold foil, a method allowing dentists to insert gold into a cavity with minimal pressure [1,10]. The foil is fabricated by annealing, a process of passing through gold making it soft and malleable. Furthermore, in the 19th century, the Dental Hospital of London opened the first clinic for the training of dentists in Great Britain. In 1859, 26 dentists met at Niagara Falls and founded the American Dental Association. In 1884, cocaine began to be used as an anesthetic in dentistry. Late, in the 21st century, novocaine was introduced [11,12].

Obviously, the extractions were practiced without anesthesia and the toothache was cured empirically with mallow poultices, with a few glasses of wine or brandy or with herbal potions [6,13,14]. Below is a remedy recommended by the Scuola Salernitana in the book “Regimen Sanitatis Salerni (Flos medicinae)”:

Regimen Salutatis Salerni (Flos medicinae)

Caput LXXX

“Si dentes serva: porrorum collidere grana, ne careas iure, cum iusqiamo simul ure. Sique per embutum fumum cape dente remotum.”

“If you want to keep your teeth, don’t waste time, burn leek seeds mixed with henbane seeds. Then direct the smoke onto the tooth with a funnel.”

With the advent of hypodermic syringes and opiates, fortunately dental anesthesia has become much effective. The development of herbal sciences has led to exploiting the properties of some plants, such as opium, obtained by engraving the not yet ripe capsules of Papaver somniferum, and collecting latex. It is known that this substance was already exploited by the Sumerians in 3500 BC to relieve toothache. Meanwhile, another US dentist, William Green Morton, was experimenting with the anesthetic virtues of ether. On 16 October 1846 he gave a public demonstration in the hall of Massachusetts General Hospital. The operation was a success: Morton made the patient breathe the fumes of an ether-soaked sponge through a glass sphere. Then the famous surgeon John Collins Warren removed a neck tumor from the volunteer, Mr. Albert Abbott, who felt no pain. In 1847 the American dentist Horace Wells was the first to experience first-hand the anesthetic virtues of nitrous oxide, or exhilarating gas, by extracting a wisdom tooth that had been painful for some time. All that is routine today in oral rehabilitation was impossible before the advent of the anesthetic. Implant-prosthetic rehabilitation, which improves the quality of life of our patients today [15,16,17,18], in the absence of anesthetic techniques would be impossible. It is necessary to consider that all pharmacological therapies today are further targeted at the clinical conditions of patients, in some cases primary pathologies make different administrations of some drugs or anesthetics different, or even the state of pregnancy [12,19]. Nowadays local anesthesia in dentistry involves blocking a small or large nerve termination through contact with the active ingredient. It is possible to divide local anesthetics into the two major categories, esters and starches. Molecules that are in common clinical use today are all included within the second category: Lidocaine, mepivacaine, articaine, and bupivacaine. Although different techniques could be distinguished between periosteal anesthesia (infiltration) and an actual nerve block, in addition to particular methods (intraligamentary, intrapulpary, and others) the route of administration is always injective. For this reason, different molecules are marketed in the form of a tube. Each tube contains the active ingredient in a predetermined quantity, possibly in association with the vasoconstrictor, in an aqueous solution. Lidocaine was synthesized in 1943 and introduced on the market in 1948, becoming the first local amidic anesthetic to be used in clinical practice, replacing procaine. The latter has been withdrawn from trade in many countries precisely because of its rapid absorption into the blood (where it could reach high levels). Mepivacaine has reduced vasodilatory properties, which makes this molecule quite effective even in the absence of vasoconstrictor. The solution for injection is therefore used in cases where the use of the vasoconstrictor is contraindicated. The articaine is the only starch with an additional ester group. The molecule therefore undergoes double metabolism, partly hepatic and partly plasma. This characteristic gives it high tissue diffusibility. It is always marketed with vasoconstrictor (adrenaline 1:100,000 or 1:200,000). Finally, bupivacaine, a molecule chemically similar to mepivacaine, but with a high liposolubility, is a long-lasting local anesthetic used at much lower concentrations. With an onset that is on average slower than the norm, it is particularly useful for long and/or long-term postoperative pain procedures [11,12].

Moreover, it is necessary to think that after the 1950s, dentistry underwent numerous and other revolutions. In 1958, a fully reclining dental chair was introduced, the first time in the history of dentistry, and from this a few years later it resulted in a technique called the “Sit down, four-handed dentistry” that has become popular. This technique improved productivity and shortened treatment time.

From the 1960s, the electric toothbrush was introduced first and subsequently the Bis-GMA (bis-pheno-glycidyl-methyl-methacrylate) was created, a resinous monomer that is the basis of current aesthetic filling materials [20], the so-called composites. In the 1990s, dentistry saw the evolution of aesthetic techniques such as tooth whitening [21].

Even today with the advent of the digital world, there is a revolution in the medical field. It is possible to carry out digital planning [22,23,24,25,26] and evaluate surgeries during the operations thanks to augmented reality. A profession that was born as a noble in ancient Egypt is subsequently diminished and separated from medicine, now it has taken a unique centrality in the general health of patients [7]. 

It might be possible that dentistry will be separated from medicine and will follow its own progress due also to technical developments. Given the fact that the number of patients with a complex medical history will increase constantly will hopefully, in contrast, integrate dentistry in the medical field. In this context, a dentist is not only characterized by their technical skills, but also by their ability to understand the medical problems of patients, allowing them to discuss medical problems with treating physicians and to further influence medical treatment.
